# Impact of Nurse Staffing Levels on Patient Fall Rates: A Retrospective Cross-Sectional Study in General Wards in Japan

**DOI:** 10.3390/healthcare13010088

**Published:** 2025-01-06

**Authors:** Mutsuko Moriwaki, Masayuki Kakehashi, Kenshi Hayashida, Masato Koizumi, Hiromasa Horiguchi

**Affiliations:** 1 Quality Management Center, Institute of Science Tokyo Hospital, 1-5-45 Yushima, Bunkyo-ku, Tokyo 113-8510, Japan; 2 Department of Clinical Data Management and Research, Clinical Research Center, National Hospital Organization Headquarters, 2-5-11 Higashigaoka, Meguro-ku, Tokyo 152-8621, Japan; 3 Graduate School of Biomedical and Health Sciences, Hiroshima University, 1-2-3 Kasumi, Minami-ku, Hiroshima 734-8553, Japan; 4 Department of Medical Informatics and Management, University Hospital, University of Occupational and Environmental Health, 1-1 Iseigaoka, Yahatanishi Ward, Kitakyushu 807-8556, Japan

**Keywords:** hospitalization, routinely collected health data, workforce, hospitals

## Abstract

**Background**: Falls are common adverse events among hospitalized patients, affecting outcomes and placing a financial burden on patients and hospitals. This study investigated the relationship between nurse staffing/workload and patient falls during hospitalization. **Methods**: The patients studied were hospitalized in the general wards (excluding pediatrics and obstetrics/gynecology) of 11 National Hospital Organization institutions between April 2019 and March 2020. The data were obtained from the Diagnosis Procedure Combination Work Record and institutional fall reports. The variables used in the analyses included patient conditions, number of hospitalization cases, emergency hospitalizations, surgeries/examinations, disease composition ratio, patient attributes, hospital stay duration, hospital bed size, and nursing time per patient (day and night) on a ward-day basis. Multivariate analysis was performed to determine the effects of these factors on fall events. **Results**: A total of 36,209 ward days were analyzed, with falls reported on 2866 days (fall event rate of 9.0%). The mean nursing times per patient were 1.99 h (day) and 1.47 h (night). The nursing time per patient in the fall group compared to the non-fall group showed an odds ratio of 1.19 (*p* < 0.01) during day shifts and 0.17 (*p* < 0.02) during night shifts. An increase in nursing time per patient during the night was associated with fewer fall events, whereas during the day, increased nursing time appeared to contribute to more falls. Common background factors that increased nurse staffing and patient falls simultaneously could be suggested to exist during the day. **Conclusions**: Increased nursing time was correlated with reduced fall incidence, indicating the need for policy improvements in nurse staffing practices in Japan to enhance patient safety and outcomes. Further research is needed to accumulate evidence reflecting policies regarding nurse staffing.

## 1. Introduction

Falls are one of the most common adverse events among hospitalized patients. In-patient falls affect patient outcomes and place a significant financial burden on patients and hospitals. It has been reported that 700,000–1,000,000 patients experience falls annually in the United States [1]. Reportedly, aging significantly affects falls [2,3]. Japan’s population is rapidly aging. Correspondingly, the number of hospitalized patients is expected to increase [4].

There is increasing evidence of a relationship between nurse staffing and patient outcomes, especially in Europe and the United States [5,6,7]. Specifically, it has been shown that allowing nurses to spend more time at the bedside of patients reduces hospital infection rates, improves glycemic control [8], lowers rehospitalization rates [9], and decreases patient mortality [9,10,11,12]. Furthermore, studies conducted in the United States have reported that reducing the number of patients per nurse decreases mortality, hospital stay duration, and rehospitalization rates contributed to cost reduction [13]. Therefore, appropriate nurse staffing is an essential factor that affects patient outcomes.

California, which has a legal limit on the number of patients a nurse can take in a day, has a lower patient mortality rate than states without such a limit. Previous studies have shown that policy decisions ensure safer staffing and patient safety in medical institutions [14,15,16]. Nurse staffing is a critical issue in ensuring patient safety. In Japan, under the influence of government policies, a large amount of medical information has been accumulated rapidly, which has enabled database research in nursing. However, few studies have examined the impact of nurse staffing on patient outcomes in Japan [17,18]. Thus, policy decisions regarding nurse staffing in Japan have been made based on scarce evidence.

In Japan, nurse staffing must be carried out as stipulated by the National Health Insurance (medical service fee system) in addition to the minimum standards required by the Medical Care Act. In 2006, a patient-to-nurse ratio system was established as a criterion for medical service fees, and the number of nurses was evaluated as a measure of remuneration. The standards for nurse staffing stipulated in the medical service fee system are set at basic hospitalization fees. The patient-to-nurse ratios in general wards are 7:1, 10:1, 13:1, and 15:1, and medical institutions must adopt one of these levels. For example, “7:1” indicates one or more nursing staff caring for seven hospitalized patients per day, meaning that nurses can be staffed at a ratio of 7:1 on an average daily. Specifically, this allows nurses to be staffed at a patient-to-nurse ratio of approximately 5–6:1 during the day shift and 14:1 during the night shift. They can be staffed unevenly each day according to the patient’s conditions and the workload in the ward. However, from a managerial perspective, it is challenging for medical institutions to allocate more nurses than the standards set by the adopted basic hospitalization fee. It is unclear whether the necessary number of nurses is assigned to patients because nurse staffing depends on the fee structure of medical service reimbursements.

Ward nurses in Japan perform a wide range of duties, including patient observation, drug administration, patient transport to surgery/examination rooms, treatment, and assistance with daily living (transfer, cleanliness care, and meal assistance). Thus, there is a significant difference in workload between daytime and nighttime nurses, including patients’ treatment plans and physical conditions. Many medical institutions in Japan have adopted team nursing methods that allow nurses to provide daily nursing care by complementing each other on a ward basis. However, this could lead to situations in which nurses cannot respond immediately to patient requests. Therefore, nursing administrators must consider patient safety from the perspective of the number of patients cared for by a nurse and the workload in the ward (i.e., the number of events in each ward, such as surgery, treatment, and patient deaths).

Nursing managers struggle to assign nurses daily based on patient outcomes, nursing experience, ward workload, and management challenges [19]. The adequacy of the methods for measuring nurse staffing and workload is unclear [20], and there is no consensus regarding the appropriateness of the methods used [21]. Ward workflow affects the workload, with patient admissions, discharges, and transfers requiring additional nursing time. There is a need to consider the ward level; however, this information is lacking. This observational study aimed to clarify the relationship between ward nurse staffing/workload and patient falls during hospitalization from the ward’s perspective.

## 2. Materials and Methods

### 2.1. Study Design

This was a retrospective cross-sectional study.

### 2.2. Study Population

**Figure 1:**
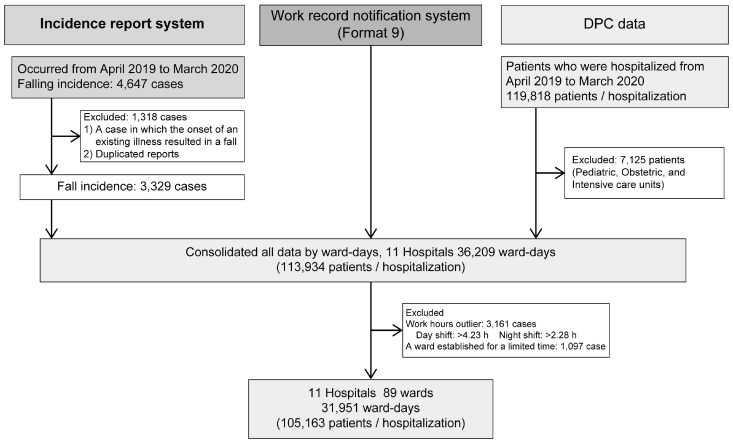
The data used for the analysis were created by combining the number of in-patients (including their characteristics), the number of nurses on staff, and the number of adverse events for each ward in each hospital on each day. These data were obtained from the DPC data, the Work Record Notification System, and the Incident Report System, respectively.

Eleven National Hospital Organization (NHO) hospitals consented to participate in this retrospective cross-sectional study, which was conducted between April 2019 and March 2020. Patients hospitalized in their general wards between April 2019 and March 2020 were examined, excluding those in critical care wards, such as the intensive care unit and high-care units, obstetrics/gynecology, and pediatric wards. The number of target patients, fall events, and working hours of nurses were aggregated and consolidated on a ward-day basis (Figure 1).

The NHO was established to manage national hospitals, and it operated 140 hospitals as of March 2020. These include general acute and specialized long-term care hospitals, 80 of which have introduced a Diagnosis Procedure Combination (DPC)-based payment system. All NHO hospitals submit administrative claims data, including DPC data, to the Medical Information Analysis databank managed by the NHO headquarters.

### 2.3. Data Sources

This study used the DPC database, work record data (Format 9), and adverse event reports (limited to falls) collected at each hospital.

### 2.4. DPC

The DPC system was introduced nationwide in acute-care hospitals in Japan in 2003. DPC is a patient classification system developed in Japan for acutely hospitalized patients. In 2003, its operation began as a comprehensive payment system by the Ministry of Health, Labor, and Welfare. It has been used for acute hospitalization care and the allocation of medical resources. Medical information related to the medical care processes of the participating hospitals has been reported to the government. Many retrospective observational studies have used DPC data [22,23]. A prior study examined the accuracy of the database [24].

As of 2021, 1755 medical institutions have been registered with the DPC system, corresponding to approximately 54% of acute hospital beds, excluding beds for infectious diseases and long-term care. DPC data used for DPC payments include the sex and age of the patient, information on clinical procedures performed, the status of hospitalization/discharge, and medical information, such as diagnosis, surgery, treatments performed, and prescribed/administered medications. The diagnosis was recorded by the attending physician using the codes of the International Classification of Diseases, 10th Revision (ICD-10). It includes six categories, which are coded as “main diagnosis”, “admission-precipitating diagnosis”, “most resource-consuming diagnosis”, “second most resource-consuming diagnosis”, “comorbidities present at the time of admission”, and “conditions arising after admission”. All procedures performed during hospitalization were recorded according to the Japanese Medical Service Fee Schedule.

Additionally, the “Severity of a Patient’s Condition and the Extent of a Patient’s Need for Medical/Nursing Care (SCNMN)” index was compiled from the DPC database. This index was developed in Japan to measure the nursing services needed by hospitalized patients and is divided into three types according to the hospital bed type: intensive care units, high-care units, and general wards. This index consists of Item A (seven items), which evaluates the status of monitoring and treatment; Item B (seven items), which assesses activities of daily living (ADL) and other conditions of patients; and Item C (seven items), which evaluates the implementation status of medical care related to surgery and emergency medicine. The evaluation items of the SCNMN index are recorded daily by nurses, and they also serve as a database that shows the daily conditions of patients [25].

The work records (Format 9) contain the daily working hours of each nurse in the ward. In Japan, the basic hospitalization fees charged by medical institutions must meet government regulations, and their criteria should include the evaluation of actual working hours. Working hours were recorded using a nationwide standardized input system and reported to the Ministry of Health, Labor, and Welfare.

### 2.5. Adverse Event Reports

In Japan, adverse event reporting is requested under the Ordinance for Enforcement of the Medical Care Act. Reporting is mandatory in some medical institutions, such as universities and national hospitals. Adverse event reporting was mandatory at the medical institutions targeted in this study. Information on in-hospital falls used in this study was obtained from the adverse event reports collected at each medical institution.

### 2.6. Outcomes

This study defined the ward days on which falls occurred as the fall group. The primary outcomes in this study adhered to ward-day levels. Falls were defined using Gibson’s definition of falls, which states “unintentionally coming to the ground or some lower level other than as a consequence of sustaining a violent blow, loss of consciousness, sudden onset of paralysis as in stroke or an epileptic seizure” [26]. We excluded falls clearly associated with the onset of an existing illness (such as a fall due to the onset of myocardial infarction) and falls outside hospital care (such as a fall that occurred while staying outside the hospital) based on the content of the cases.

### 2.7. Patient Variables

For variables that indicated workload on ward days, we obtained data on the sex and age distribution (sociodemographic distribution) of patients hospitalized in the ward on that day, the clinical state of patients, the status of nursing care, and the patient’s physical function. This study used values obtained by dividing the number of applicable patients by the total number of patients in the ward on that day. Age and sex were used to assess sociodemographic status. Regarding the clinical state of the patients, we obtained data on the use of psychiatric drugs, hypertension, osteoporosis, presence or absence of anemia, status of comorbidities during hospitalization, and presence or absence of emergency hospitalizations. The scores for comorbidities during hospitalization were calculated using the Quan version [27] of the Charlson comorbidity index [28]. The daily working hours of each nurse during the day (8 h) and night (16 h) were recorded in Format 9 for each ward. Nursing time per patient during the day and night was calculated by adding the values (hours) for each day and dividing them by the number of patients.

Item A of the SCNMN index was used to assess the status of nursing care (highly specialized nursing care, including monitoring and treatment). Item B was used for patient functional classes, such as ADL, and Item C was used for medical management, such as medical treatment related to surgery and emergency care [25]. These variables indicate patients’ conditions, status of nursing care, and workload on ward days.

### 2.8. Statistical Analyses

The data were treated as distinct observations for each ward and day, i.e., observation unit is a ward of each day. Because nurse staffing is the attribute of ward of each day, it seemed natural to adopt a ward of each day as an observation unit. The primary outcome variable is the occurrence of falling within a specific ward on a given day. Initially, we performed descriptive statistics. Subsequently, outliers, characterized by deviations greater than 2 SD from the mean, were identified in relation to both patient count per ward and nursing time across day/night shifts. Such variables were excluded. Subsequent steps involved pairwise comparisons for all variables, utilizing the Mann–Whitney *U* and chi-square tests. Logistic regression analysis was conducted with the occurrence of falls as the dependent variable, incorporating only those variables displaying a significant correlation with fall incidence as independent factors. We employed IBM SPSS Statistics for Windows, version 29 (IBM Corp., Armonk, NY, USA) for all statistical analyses, setting statistical significance at *p* < 0.01 or 0.05.

## 3. Results

We collected data on 36,209 ward days in 11 facilities on a day-and-ward basis. Outliers with a nursing time per patient of >4.23 h during the day shift and >2.28 h during the night shift, as well as a ward established for a limited time, were excluded from the analyses, resulting in 31,951 ward days and 105,163 patients (Figure 1). Fall events were observed on 2866 ward days but not on 29,085 ward days. The mean age of the target patients was 71.1 years (SD, 15.2), with males accounting for 52.3%, and the fall event rate in the wards was 9.0%. The mean nursing time per patient was 1.99 h (SD, 0.59) during the day shift (8 h) and 1.47 h (SD, 0.24) during the night shift (16 h) (Table 1).

Regarding the number of events in the fall group by ward, 93% of wards had one event per day (2684 ward-days), and 182 ward-days (7.0%) had two or more events per day (Supplementary Table S1). The mean nursing time per patient (SD) during the day shift was 1.99 h (0.60) in the non-fall group and 2.01 h (0.58) in the fall group (*p* < 0.01). Similarly, the mean nursing time per patient (SD) during the night shift was 1.47 h (0.24) in the non-fall group and 1.46 h (0.23) in the fall group (*p* < 0.01) (Table 2).

These results show that compared with the non-fall group, the nursing time per patient in the fall group was longer during the day shift and shorter during the night shift. The proportion of patients aged ≥65 years was 71.4% (13.2) in the non-fall group and 73.2% (12.1) in the fall group (*p* < 0.01). The rate of in-hospital death was 0.17% (0.65) in the non-fall group and 0.21% (0.70) in the fall group (*p* < 0.01) (Table 2). Furthermore, the wards in the fall group had a higher proportion of patients who received ECG monitor management, respiratory care (except for only sputum aspiration), and special treatment related to critical care (Table 3).

Logistic regression analysis showed that compared to the non-fall group, nursing time per patient in the fall group had an odds ratio (OR, the exponential of the partial regression coefficient β, exp(β)) of 1.19 (*p* < 0.01) during the day shift and an OR of 0.71 (*p* < 0.01) during the night shift, which indicates that an increase in nursing time per patient at night reduced fall events; in contrast, an increase in nursing time per patient during the day increased fall events. Moreover, hospital deaths were associated with falls (OR = 1.08, *p* < 0.01) (Table 4).

## 4. Discussion

In Japan, the staffing of nurses for patients is determined based on the medical service fee system. However, in practice, the Medical Care Act allows nursing administrators to assign nurses to wards with high workloads depending on their daily situation. Therefore, the number of staff per ward differed from that reported in the medical service fee system. Moreover, Spetz et al. pointed out a significant difference between staffing at the unit and hospital level [29,30]. Based on the findings of these previous studies and for the first time in Japan, we investigated the factors that affect patient falls in hospital wards from the perspective of nursing time per patient and workload of day/night shifts to clarify the relationship between nurse staffing and falls, which are patient outcomes.

Regarding patient falls in hospital wards, an increase in nursing time per patient during the night shift reduced the occurrence of fall events. In contrast, an increase in nursing time per patient during the day shift increased the occurrence of fall events. Ward nurses in Japan perform a wide range of duties, including patient observation, drug administration, patient transport to surgery/examination rooms, treatment, and assistance with daily living (transfer, cleanliness care, and meal assistance). Thus, their workload varies depending on the patients’ treatment plans and conditions, and nurses have a significant workload difference between the day and night shifts. Therefore, in the staffing of day shift nurses, an increase in nursing time per patient might be allocated to performing duties such as surgery and treatment, leaving considerably limited time for direct patient observation, which allows nurses to respond immediately to patients when needed. However, night-shift nurses have more time for direct patient observation because of their small workload during surgery and treatment. With low patient activity at night, an increase in nursing time per patient during the night shift may reduce the occurrence of falls. Previous studies have shown that the greater the number of nurses, the lower their fall rates [31]. A Japanese study also showed that the higher the number of nurses assigned per bed, the lower the risk of in-hospital fractures [32]. Our findings regarding night shifts are consistent with those of previous studies [33]. However, it has been pointed out that inconsistent results have been obtained in Japanese studies on in-hospital falls that influence in-hospital fractures [21]. This indicates the need for further research on nurse staffing levels and patient outcomes in Japan.

In Japan, nurses are staffed to the extent that they do not conflict with the standards of the Medical Care Act from an economic point of view, such as labor costs, and none of the hospitals have an abundance of nurses. Therefore, the number of day-shift nurses is insufficient to increase the time spent directly caring for patients. The current nurse staffing during day shifts may not adequately ensure patient safety.

This study was conducted based on the hypothesis that the occurrence of falls, which is greatly affected by patient factors, may also be affected by the ward’s environment. Falls have been reported to be strongly influenced by patient characteristics, and patient age and ADL (especially the state of transfer) significantly affect the occurrence of falls [2,3,34]. In the present study, we performed analyses on a ward-day basis, showed the proportion of patients at high fall risk among ward patients, and adjusted for patient conditions and medical care processes on a ward basis. As a result, the “occurrence of death in hospital wards was shown to affect falls, which suggests that the ward environment (such as workload) may increase the risk of falls, regardless of the patient’s condition. Specifically, when an event such as death occurs in a ward, the nurse in charge of the patient and other nurses support each other, shouldering an increased workload. Therefore, there is a tendency for other patients to be temporarily under-monitored. Patrician et al. [31] reported that “Fundamental questions remain, including how ward-level patient care factors, beyond the structural indicators, interact to alter patient care outcomes and hospital-level performance”. Furthermore, patient outcomes are affected by individual patient factors, the number of staff assigned, the skill mix, and the workload [35,36]. In other words, ward factors may appear as a collection of nurses, patients, and individual characteristics, and it is necessary to capture them in the hospital ward environment [37].

He et al. [38] reported that the rate of patient falls was inversely associated with total nursing hours per patient day (HPPD) in their study in the USA. They also noticed the influence of seasonality: the total HPPD tended to be lower during January–March when falls were more likely to happen. Wong et al. [39] reported that patients’ fall rate correlated positively with the nurse-to-patient ratio based on a study of hospitals in China, additionally reporting a negative correlation with the proportion of nurses with less than 5 years of work experience. Kim et al. [40] found that the HPPD by registered nurses was a significant factor influencing in-hospital falls in their study in Korea. The results of Seeherunwong et al. [41] in a study of psychiatric hospitals in Thailand also showed an association between inpatient falls and the nurse-to-patient ratio, although it was not statistically significant. Nurse staffing is an influential factor, along with other factors whose associations are not yet fully clarified. Further studies are required to identify which background factors are important in predicting and preventing patient falls.

In Japan, nurses perform a wide range of duties, and nurse staffing enables flexible changes depending on the daily workload and patient conditions. In such a nursing system, environmental factors for patients and wards should be considered to prevent adverse events. This study suggests that adverse events, including falls, which are significantly affected by patient characteristics, can be controlled by improving the ward environment, including nurse staffing.

### Limitation, Strengths, and Future Studies

This study targeted acute care medical institutions with general hospital beds, and the analyses were performed by adjusting for patient background. However, differences in patient background that could not be adjusted for in the DPC data may have resulted from hospital factors. Furthermore, hospital-specific circumstances, such as structural issues and organizational characteristics of hospitals, may have affected the outcomes.

The staffing hours of the nurses used in this study were obtained from Format 9. The data describe the ward working hours within the total hours stipulated in the data submitted to the Ministry of Health, Labor, and Welfare to assess whether facility standards for medical service fees are met. Therefore, overtime was not considered in this study. Moreover, although the staffing status of nurse aides is thought to have an effect, this was not reflected in this study because of data acquisition limitations.

However, obtaining accurate working hours for nurses in Japanese wards is time-consuming. This study used the most detailed available information. Furthermore, previous studies that clarified the relationship between nurse staffing and patient outcomes in Japan used the ratio of the number of hospital beds to the full-time equivalent number of nurses based on information in the hospital report (the Annual Report for Functions of Medical Institutions), which reports the status of nurse staffing to the government annually [18,32,42]. Information on nurse staffing is currently available in these databases. However, in practice, the number of nurses staffed in hospital wards can be determined based on the daily workload and patient conditions within the framework of the system. Thus, it is desirable to clarify patient outcomes based on the staffing status, which reflects the actual situation of the ward to a certain extent. This study is the first to demonstrate the staffing status in hospital wards at the time level. Furthermore, this study is a multi-institutional study that utilized data from DPC records, incident reports, and nurse working hour data—datasets that are typically difficult to link. The insights gained from the vast amount of daily data are considered highly valuable.

In the future, it will be necessary to develop systems that enable nursing managers to monitor, in real time, the relationship between nursing hours and adverse events such as falls and injuries in actual clinical settings, as well as to flexibly adjust nurse staffing accordingly.

## 5. Conclusions

In Japan, medical expenses are increasing annually owing to the rapidly aging population. As a countermeasure, the government has focused on nurses with a high personnel ratio among health professionals. It has adopted policies that have reduced the number of nurses, such as increasing the patient-to-nurse ratio in the basic hospitalization fee system and tightening standards. In the present study, increased nursing time per patient during night shifts was associated with decreased fall events, which suggests that current nurse staffing during day shifts is insufficient to ensure patient safety. In other words, if day shift nurses are sufficiently staffed according to workload, the results obtained for night shifts may also be seen for day shifts. However, this is difficult because of systematic or hospital management issues. Evidence regarding the relationship between nurse staffing and patient outcomes in Japan is exceptionally scarce. Further research is needed to accumulate evidence reflecting policies regarding nurse staffing.

In countries and medical institutions where team-based nursing methods are adopted, nurses provide patient care by complementing each other throughout the ward. In such cases, it is necessary to examine nurse staffing by comprehensively evaluating the nurse workload and patient background from the perspective of individual patients and nurse- and ward-related factors. If further research accumulates strong evidence of a relationship between patient outcomes and workload at the ward level, information such as nurse staffing and patient outcomes, including in-hospital fractures and workload, may be available at the ward level, which may lead to the development of medical information systems that can store and provide relevant data. With this system, the information necessary for management can be presented quantitatively to the head nurse in real time, supporting decision making regarding day-to-day ward management.

## Figures and Tables

**Table 1:** Characteristics of the hospital ward (N = 31,951).

	Ward	Number of Units (Ward-Days)	Fall Event Rate ※1	Total Number of Patients/Hospitalization	Total Number of Patients	Age		Man		Surgical Patient		Nursing Time per Patient			
												Day Shift (8 h)		Night Shift (16 h)	
						Mean	SD	Number	Rate ※2	Number	Rate ※2	Mean	SD	Mean	SD
Hosp1	5	1826	8.38	4249	6066	73.11	14.9	2179	51.3	2114	49.8	1.96	0.61	1.38	0.19
Hosp2	8	2908	11.28	7946	10,106	72.56	16.1	4059	51.1	4404	55.4	2.00	0.53	1.59	0.19
Hosp3	9	3241	6.54	6943	9323	71.54	15.1	3637	52.4	3944	56.8	1.97	0.51	1.45	0.26
Hosp4	6	2158	9.73	5883	7969	70.96	15.8	2842	48.3	3044	51.7	2.08	0.53	1.55	0.23
Hosp5	5	1771	10.33	3842	5177	74.24	14.1	2088	54.3	1823	47.4	2.21	0.66	1.52	0.25
Hosp6	9	3261	10.55	7014	10,073	70.57	15.3	3826	54.5	3599	51.3	1.95	0.47	1.49	0.19
Hosp7	11	3993	5.53	7876	12,105	68.65	15.9	4099	52.0	4341	55.1	2.06	0.66	1.47	0.24
Hosp8	9	3084	8.98	7406	11,227	70.17	15.7	4069	54.9	3873	52.3	2.37	0.58	1.61	0.24
Hosp9	8	2926	11.28	6305	9781	72.81	14.1	3330	52.8	3250	51.5	2.01	0.48	1.26	0.17
Hosp10	12	4331	8.77	9642	14,847	69.74	14.4	4864	50.4	4811	49.9	1.37	0.20	1.39	0.21
Hosp11	7	2452	9.30	6109	8489	71.00	15.0	3335	54.6	3494	57.2	2.34	0.53	1.53	0.25
Total	89	31,951	8.97	73,215	105,163	71.13	15.2	38,328	52.3	38,697	52.9	1.99	0.59	1.47	0.24

※1 Fall event rate: Number of events Number of units where occurrence (word-days)/number of units. ※2 Percentage of Total number of patients.

**Table 2:** Characteristics of ward-days (N = 31,951).

	Non-Fall Group N = 29,085		Fall Group N = 2866		*p* *1
	Mean	SD	Mean	SD	
**Nursing time per patient**					
Day shift (8H), h	1.99	0.60	2.01	0.58	0.05
Night shift (16H), h	1.47	0.24	1.46	0.23	0.02
**Patients background**					
65 years old and over, %	71.40	13.20	73.18	12.19	<0.01
Man, %	57.63	14.75	58.15	13.43	0.53
Discharge, %	6.98	4.96	6.97	4.91	0.93
Admission, %	6.15	5.38	6.16	5.26	0.56
In-hospital death, %	0.17	0.65	0.21	0.70	<0.01
Sedative hypnotics, %	13.16	7.87	13.31	7.88	0.30
Psychotropic, %	9.95	7.04	10.38	7.41	0.03
Hypertension, %	33.12	16.59	34.11	16.94	<0.01
Osteoporosis, %	4.66	5.88	4.65	5.22	0.03
Anemia, %	14.43	9.93	14.41	9.53	0.50
CCI score	1.34	0.49	1.37	0.47	<0.01
Emergency hospitalization, %	1.01	1.72	0.99	1.68	0.82
Surgery, %	1.48	2.79	1.43	2.71	0.63
Injection, %	25.85	12.83	26.84	12.76	<0.01

*1 Mann–Whitney U test.

**Table 3:** Characteristics of patients in wards from the perspective of SCNMN (N = 31,951).

	Non-Fall Group N = 29,085		Fall Group N = 2866		*p* *1
	Mean	SD	Mean	SD	
**<Monitoring and treatment>**					
Wound treatment (excluding treatment of pressure ulcer), %	4.37	6.05	4.04	5.71	0.02
Treatment of pressure ulcers, %	0.30	0.98	0.30	0.97	0.81
Respiratory care (except for only sputum aspiration), %	8.95	7.84	9.39	8.19	0.02
Management of three or more intravenous lines at the same time, %	5.57	6.86	5.44	6.63	0.96
ECG monitor management, %	19.30	16.82	19.87	16.79	0.02
Syringe driver management, %	1.96	3.38	2.07	3.54	0.20
Management of blood transfusion and blood product, %	1.82	3.00	1.73	2.82	0.47
Professional treatment, %	25.85	13.85	25.47	13.20	0.47
Use of antineoplastic agents (injection only), %	1.92	3.73	1.81	3.51	0.68
Management of oral administration of antineoplastic agents, %	1.79	2.63	1.70	2.49	0.16
Use of narcotics (injection only), %	2.15	3.14	2.10	3.08	0.60
Internal use of narcotics, application, management of suppositories, %	1.86	3.16	1.76	3.11	0.08
Radiation therapy, %	1.53	3.18	1.62	3.26	0.16
Immunosuppressant management, %	9.22	9.46	9.25	8.82	0.17
Use of pressor agent (injection only), %	1.64	2.75	1.65	2.76	0.74
Use of antiarrhythmic agent (injection only), %	0.38	1.16	0.35	1.13	0.28
Use of continuous infusion of antithrombotic embolic drug, %	3.17	4.05	3.29	4.15	0.11
Drainage management, %	5.94	7.15	5.80	7.10	0.31
Treatment in a sterile treatment room, %	1.16	5.19	0.84	4.50	<0.01
**<Patients’ functional state>**					
Turn over (Partly assisted), %	28.23	18.60	27.39	17.33	0.38
Turn over (Fully assisted), %	16.68	10.36	17.40	10.54	<0.01
Transfer (Partly assisted), %	32.96	15.88	32.52	14.64	0.88
Transfer (Fully assisted), %	12.28	8.90	13.27	9.35	<0.01
Oral care,%	44.27	15.27	45.73	15.27	<0.01
Meal intake (Partly assisted), %	28.08	15.52	27.46	14.48	0.35
Meal intake(Fully assisted), %	10.59	9.09	11.34	9.59	<0.01
Personal dressing (Partly assisted), %	26.87	13.28	27.36	13.09	0.03
Personal dressing(Fully assisted),%	21.23	13.58	22.75	14.46	<0.01
No able to receive directions on medical care and treatment, %	19.11	18.50	21.80	20.23	<0.01
Engaged in dangerous behavior, %	9.77	9.40	10.59	10.15	<0.01
**<Surgery and emergency care>**					
Craniotomy (within 7 days from the day of surgery), %	0.11	0.64	0.12	0.68	0.21
Thoracotomy (within 7 days from the day of surgery), %	0.10	0.59	0.11	0.63	0.52
Laparotomy (within 4 days from the day of surgery), %	0.51	1.45	0.47	1.31	0.61
Bone surgery (within 5 days from the day of surgery), %	1.76	4.78	1.64	4.61	0.06
Thoracoscopic/laparoscopic surgery (within 3 days from the day of surgery), %	1.04	2.39	1.01	2.31	0.45
General anesthesia/spinal anesthesia surgery (within 2 days from the day of surgery), %	3.01	4.43	2.97	4.42	0.86
Special treatment related to critical care (within 2 days from the day of treatment), %	1.32	2.93	1.53	3.18	<0.01
Percutaneous endovascular treatment, %	0.39	1.28	0.45	1.45	0.09
Treatment such as percutaneous myocardial ablation, %	0.34	1.53	0.34	1.48	0.71
Invasive gastrointestinal treatment, %	0.60	1.83	0.74	2.12	<0.01
Eye surgery, %	0.21	1.17	0.19	1.06	0.38

*1 Mann–Whitney U test.

**Table 4:** Results of logistic regression analysis for accidental fall (N = 31,951).

	B	OR	OR 95% CI		*p*
			Lower	Upper	
Nursing time per patient:day shift (8H), h	0.17	1.19	1.09	1.29	<0.01
Nursing time per patient:night shift (16H), h	−0.34	0.71	0.53	0.95	0.02
In-hospital death, %	0.08	1.08	1.02	1.14	<0.01
Wound treatment (excluding treatment of pressure ulcer), %	−0.02	0.98	0.98	0.99	<0.01
Treatment in a sterile treatment room, %	−0.02	0.98	0.97	0.99	<0.01
Oral care, %	0.01	1.01	1.00	1.01	<0.01
Osteoporosis, %	−0.01	0.99	0.98	1.00	0.01

Note: Adjusted for number of patients in hospital (%), hospital nest.

## Data Availability

The datasets generated and/or analyzed in the current study are not publicly available because of contracts with hospitals providing data to the database. The data are available from the coauthor (H.H.) upon reasonable request.

## Supplementary Materials

The following supporting information can be downloaded at https://www.mdpi.com/article/10.3390/healthcare13010088/s1. Table S1: Number of events occurring by hospital ward.
