# Supplementary material for: Impact of Nurse Staffing Levels on Patient Fall Rates: A Retrospective Cross-Sectional Study in General Wards in Japan
Source: Healthcare (Basel). 2025 Jan 6;13(1):88. doi: 10.3390/healthcare13010088 (PMC11719517; doi:10.3390/healthcare13010088)
Supplement: Supplementary file 1 [file healthcare-13-00088-s001.zip › healthcare-3378277-supplementary.pdf]

Supplementary Table S1. Number of events occurring by hospital wards

N = 31,951

	Non-fall group		Fall group		Number of fall events per day*1							
					1		2		3		4	
Hospital	Number of ward-days	%	Number of ward-days	%	Number of ward-days	%	Number of ward-days	%	Number of ward-days	%	Number of ward-days	%
Hosp1	1,673	5.24	154	0.48	142	0.44	10	0.03	1	0.00	1	0.00
Hosp2	2,580	8.07	328	1.03	311	0.97	16	0.05	1	0.00	0	0.00
Hosp3	3,029	9.48	212	0.66	207	0.65	3	0.01	2	0.01	0	0.00
Hosp4	1,948	6.10	210	0.66	188	0.59	20	0.06	1	0.00	1	0.00
Hosp5	1,588	4.97	183	0.57	171	0.54	11	0.03	1	0.00	0	0.00
Hosp6	2,917	9.13	344	1.08	310	0.97	29	0.09	5	0.02	0	0.00
Hosp7	3,772	11.81	221	0.69	210	0.66	11	0.03	0	0.00	0	0.00
Hosp8	2,807	8.79	277	0.87	263	0.82	13	0.04	1	0.00	0	0.00
Hosp9	2,596	8.12	330	1.03	309	0.97	20	0.06	1	0.00	0	0.00
Hosp10	3,951	12.37	380	1.19	361	1.13	19	0.06	0	0.00	0	0.00
Hosp11	2,224	6.96	228	0.71	212	0.66	16	0.05	0	0.00	0	0.00
Total	29,085	91.03	2866	8.97	2,684	8.40	168	0.53	12	0.04	2	0.01

*1 This shows the number of falls that occurred in the ward per day.
